# Distributed Small-Step Path Planning and Detection Method for Post-earthquake Robot to Inspect and Evaluate Building Damage

**DOI:** 10.3389/fnbot.2022.915150

**Published:** 2022-08-15

**Authors:** Zhaojia Tang, Ping Wang, Yong Wang, Changgeng Wang, Yu Han

**Affiliations:** ^1^School of Intelligent Systems Engineering, Sun Yat-sen University, Guangzhou, China; ^2^Guangdong Provincial Key Laboratory of Fire Science and Intelligent Emergency Technology, Guangzhou, China

**Keywords:** building inspection, inspection robot, building damage, shooting angle, detection performance

## Abstract

Post-earthquake robots can be used extensively to inspect and evaluate building damage for safety assessment. However, the surrounding environment and path for such robots are complex and unstable with unexpected obstacles. Thus, path planning for such robot is crucial to guarantee satisfactory inspection and evaluation while approaching the ideal position. To achieve this goal, we proposed a distributed small-step path planning method using modified reinforcement learning (MRL). Limited distance and 12 directions were gridly refined for the robot to move around. The small moving step ensures the path planning to be optimal in a neighboring safe region. The MRL updates the direction and adjusts the path to avoid unknown disturbances. After finding the best inspection angle, the camera on the robot can capture the picture clearly, thereby improving the detection capability. Furthermore, the corner point detection method of buildings was improved using the Harris algorithm to enhance the detection accuracy. An experimental simulation platform was established to verify the designed robot, path planning method, and overall detection performance. Based on the proposed evaluation index, the post-earthquake building damage was inspected with high accuracy of up to 98%, i.e., 20% higher than traditional unplanned detection. The proposed robot can be used to explore unknown environments, especially in hazardous conditions unsuitable for humans.

## Introduction

**Figure 1:**
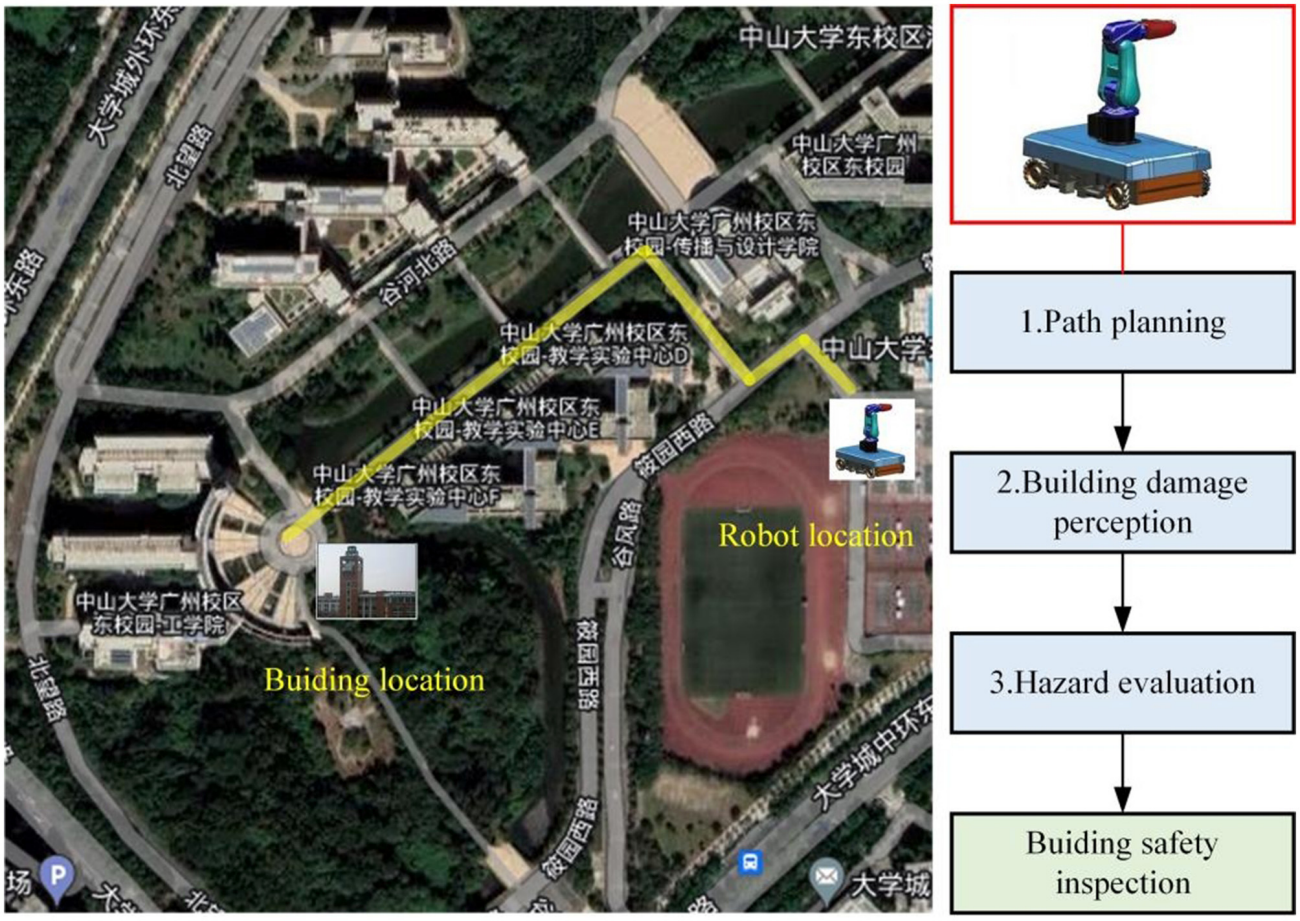
Robot designed for the post-earthquake inspection and evaluation.

A strong earthquake, which can damage the buildings that put them at risk of collapse at any time, which threatens the safety of rescue forces. Thus, inspection and evaluation post-earthquake buildings safety are crucial to devise rescue strategies and save survivors. The use of the robot with remote sensing abilities are relatively safer under harsh conditions to avoid unnecessary injury to the rescue team. Robot rapid inspection and evaluation damaged building for prompt rescue, quick response and fast alarm can avoid numerous deaths, which are quite useful during emergencies. However, the mobility of robot in a post-earthquake environment is complex and unstable owing to unexpected obstacles, uneven ground, and other unknown situations. Therefore, compared with the ordinary environment, the post-earthquake environment is much more complicated, and the robot buildings inspection needs to overcome many difficulties (Figure 1).

The first challenge for the robot used in a disastrous environment is path planning. The main obstacle for the robot to access the required area is the interference of uncontrollable road obstacles in the post-earthquake scenario (Lin et al., 2022). This involves planning a conflict-free path between the specified start and end positions. For this purpose, there are two solutions as follows: off-line planning (Morozov et al., 2018) and online planning (Lu et al., 2020). Online planning is more suitable in post-earthquake scenarios with minimal path planning based on energy-saving requirements (Quagliarini et al., 2018). Additionally, collision-free paths based on complex environments were studied (Wang and Chen, 2022). Unmanned technologies with highly variable terrain features have been reported (Bayat et al., 2018). The complex environment formed by extensive damage in dense areas of buildings (Demir et al., 2021) is not conducive to the inspection of buildings by robots. Usually, only a narrow space is available for the robot to pass. However, the traditional dynamic path planning methods might not be suitable for this purpose. Recently, reinforcement learning (RL) was fused with path planning to navigate complex disaster sites (Li et al., 2011; Zhao et al., 2018; Liu et al., 2021). It is a feasible method to plan a safe path for the robot in demanding environments with the help of RL so that robot can capture a view of the building from a suitable angle for evaluating building safety using photo processing algorithms. Additionally, robot-assisted inspection is widely used in structure monitoring (Oh et al., 2009; Murphy et al., 2011; Amhaz et al., 2016; Quintana et al., 2016; Peel et al., 2018; Cai and Mostofi, 2019; Yan et al., 2019; Lin et al., 2022). Although the background environment of these applications is simple, but they provide a feasible concept for the detection of buildings and their safety assessment under demanding environments. The difficulty in the post-earthquake environment is how the robot adapting complex shapes and irregularly distributed obstacles (Mucka, 2013; Al-Baghdadi et al., 2020; Luo et al., 2020; Su et al., 2020). It is obvious that the traditional path planning methods are difficult to meet this harsh condition.

The second challenge is how to accurately reflect the damage of the building through photos, which can be used to predict the damage caused by the further collapse of the building in post-earthquake environment. It is a common method to evaluation the safety of the building by the deformation of the building or the inclination angle with the ground (Lipecki, 2022), but the damage to the building by the earthquake can often lead to more complex deformation, which makes the post-earthquake environment building safety inspection is difficult. Usually, the maximum inclination direction of building is further collapse direction (Wu et al., 2021). Therefore, it is important to accurately reflect the maximum inclination direction of the building by adjusting the camera shooting angle.

The third challenge is that the photo method should be fast and accurate to identify the building damage after the robot obtains the picture. There are various technical solutions for detecting building deformation and other feature analyses (Wu and Liu, 2012; Shi et al., 2016; Huyan et al., 2019). Considering the complexity of demanding environments and the requirements of fast inspection and higher accuracy results, corner point detection algorithm only extracts the corner point information in the photo instead of the entire photo (Ye et al., 2021; Zhang and Sun, 2021). It is a fast technique to meet the detection requirement. However, the existed corner detection algorithms have poor detection effect on crack features with complex shapes (Hou, 2010; Zhong et al., 2010; Zheng and Lin, 2020). Actually, combining an appropriate corner point detection algorithm with robot path planning, capturing damaged buildings in complex environments, and making fast and accurate analysis and evaluation is significant to improve safety in monitoring building damage.

The rest of the article is organized as follows: In the “Methods” section, first of all, we propose a small-step path planning method with modified reinforced learning (MRL) to optimize the path of the robot to adapt to the complex and unstable environment. Second, by planning the camera angle to obtain high-quality photos for corner point detection. At last, we designed a corner point detection algorithm based on the improved Harris algorithm (H–G) for detecting tilt, displacement, cracks, and other exterior features of a building. In the “Experiment and Result” section, we confirmed the effectiveness and reliability of the proposed method for corner point detection of buildings in post-earthquake environment. In the “Discussion” section, we discuss the potential shortcomings of the proposed method along with more accurate building safety assessment detection methods for the future. The “Conclusion” section summarizes the study.

## Methods

**Figure 2:**
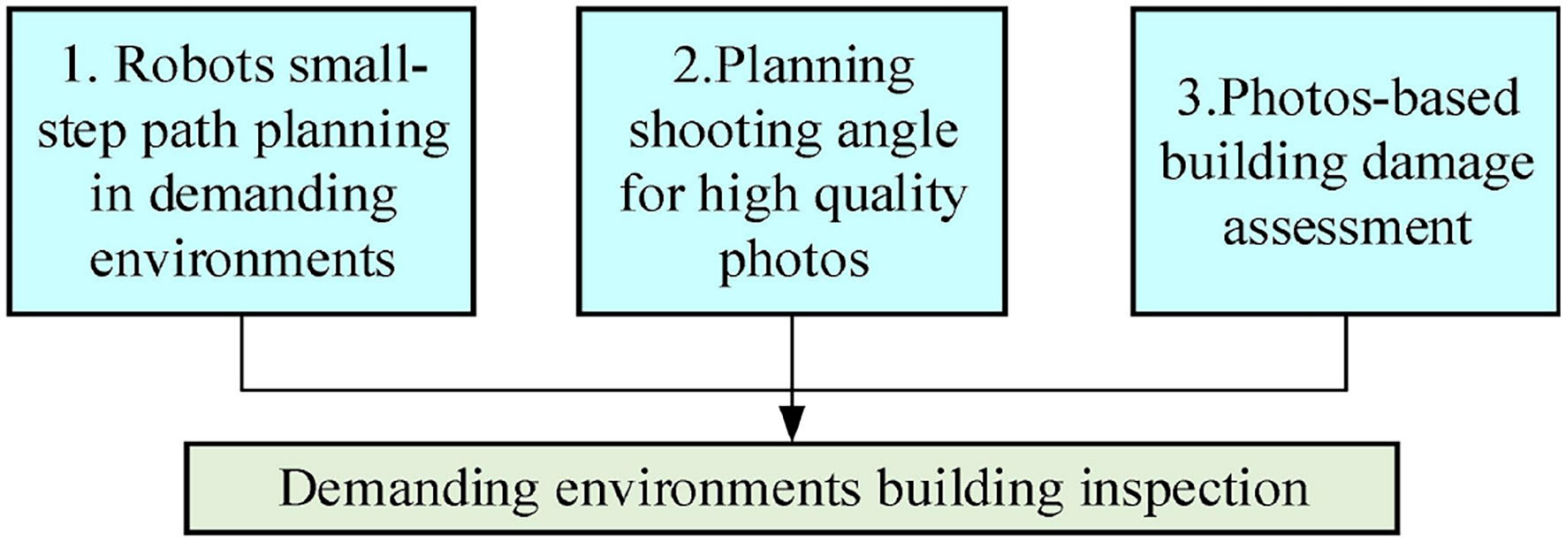
Robots utilized to inspect and evaluate the building damage for safety considerations.

When a building safety inspection is planned in ordinary environments, first, people need to arrive at the best location, which near the building, and take the building photos, then evaluate the safety of the building by detecting the corner points in photos. However, compared with ordinary environments, there are three difficulties when inspecting and evaluating building damage in post-earthquake environments: (1) People could not enter the environment to find a best location for take photo, (2) it is difficult to find a best shooting angle to reflect the inclination of the building, (3) the complex and irregular cracks appearance on the building make the plain algorithm is difficult to find the corner points on the building. For solve the difficulties: (1) we propose a small-step path planning method with modified reinforced learning (MRL) for the robot, which simulates a human's gait (small steps) to avoid obstacles in complex environment, we use robot instead of humans to work in post-earthquake environments, (2) we propose a method for shooting angle planning, that the camera on the robot can obtain a photo which is the best angle to reflects the inclination of the building, and (3) we propose an improved corner point detection algorithm for enhance the complex and irregular cracks detection accuracy. Finally, we use these three techniques to inspect the building in demanding environments, the detailed process is shown in Figure 2.

### Robot Small-Step Path Planning in Demanding Environments

**Figure 3:**
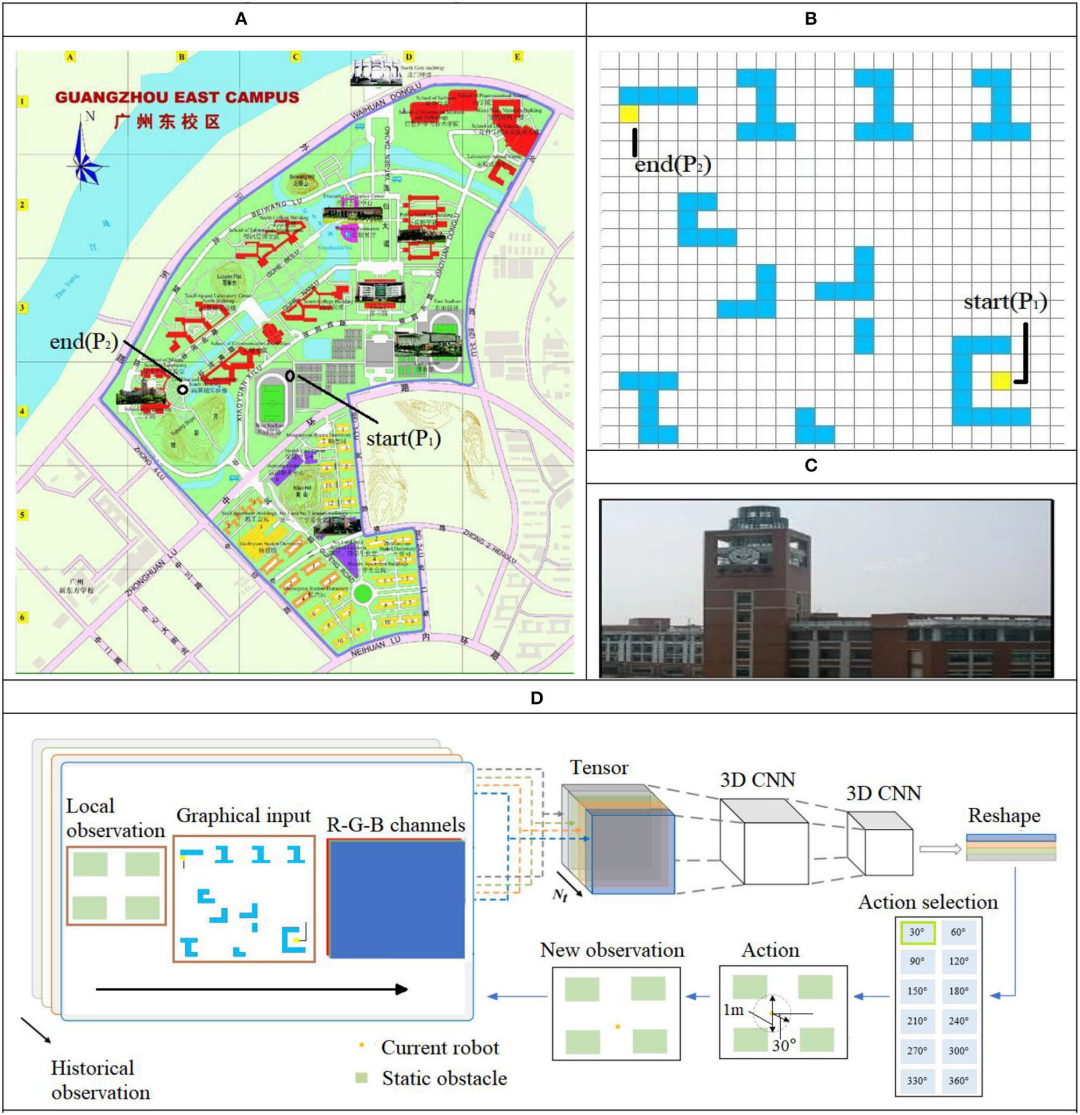
Scene description and modified reinforcement learning (MRL). **(A)** Top view of path planning environment. **(B)** Obstacle distribution. **(C)** Detection object. **(D)** Process of MRL.

Figure 3 shows the process of post-earthquake robot inspection; in Figure 3A, points P_1_ and P_2_ are the starting and ending positions of the robot, respectively. Figure 3B shows the distribution of obstacles along the path. Figure 3C shows the object photographed by the robot from point P_2_.

In a post-earthquake environment, due to the complex distribution of obstacles, people can only walk with a small-step size (0.5 m), at the same time, approach the target position by continuously adjusting the walking direction. Inspired by human behavior, we design an algorithm for the robot to avoid obstacles and walking in the shortest distance in complex environments, as shown in Figure 3D; RL helps the robot to plan the path using small moving steps to ensure the path planning to be optimal in a neighboring safe region so that the robot can avoid obstacles within a diameter of 1.0 m in steps of 0.5 m while moving. After a step-by-step iteration, the optimal walking path between the detected target and the building was calculated.

In Figure 3D, MRL combines the image information (in 2D) of the local environment and the overall environment using multiple photos consisting of three dimensions as the input for the neural network. The weights of the neural network are used to learn the features and provide the basis for decision making for obstacle avoidance in the output layer. The stepwise rolling optimization strategy in this study has a planning radius of 0.5 m. The forward direction of the robot within a step of 0.5 m is given in the output layer of the network (at 30° intervals for the path direction selection) after one iteration. After obtaining the new position, the new position information replaces the previous local position information as the input to the network for the next iteration. This cyclic process eventually provides the results for multiple local small-step path optimization to cover the entire environment. In the given environment, the walking path of the robot via small steps and MRL updates the moving direction, thereby enabling the robot to avoid unknown disturbances. The algorithm is shown Algorithm table:

**Algorithm 1:** Deep Q-learning with experience replay.

Initialize replay memory *D* to capacity *N*
Initialize action–value function *Q* with random weights θ
Initialize target action–value function $\hat{Q}$ with weights θ^−^ = θ
**For** episode = 1, *M* **do**
Initialize sequence *s*_1_ = {*x*_1_} and preprocessed sequence ∅_1_ = ∅(*s*_1_)
**For** *t* = 0.5, *T* **do**
With probability ε, select a random action *a_*t*_*
Otherwise select *a_*t*_* = argmax*_*a*_Q*(∅(*s_*t*_*), *a*; θ)
Execute action *a_*t*_* in the emulator and observe reward *r_*t*_* and image *x*__*t*+ _1_
Set *s*__*t*_+1_ = *s_*t*_, a_*t*_*, *x*__*t*+_1_, and preprocess ∅_*t*+1_ = ∅(*s*__*t*_+ 1_)
Store transition (∅_*t*_, *a_*t*_, r_*t*_*,∅_*t*+1_) in *D*
Sample random minibatch of transitions (∅_*j*_, *a_*j*_, r_*j*_*, ∅_*j* + 1_) from *D*
$Sety_{j}=\left\{ \begin{array}{l} \;r_{j\;}if\;episode\;terminates\;at\;step\;j+1\\ r_{j}+\gamma \underset{a^{{}^{\prime }}}{\text{max}}Q(\emptyset_{j+1},a^{{}^{\prime }};\theta^{-})\;otherwise \end{array}\right.$
Perform a gradient descent step on [*y_*j*_* – *Q*(∅_*j*_, *a_*j*_*; θ)]^2^ with respect to the network parameters θ
Every *C* steps reset $\hat{Q}$ = *Q*
**End For**
**End For**

### Planning Shooting Angle for High-Quality Photos

**Figure 4:**
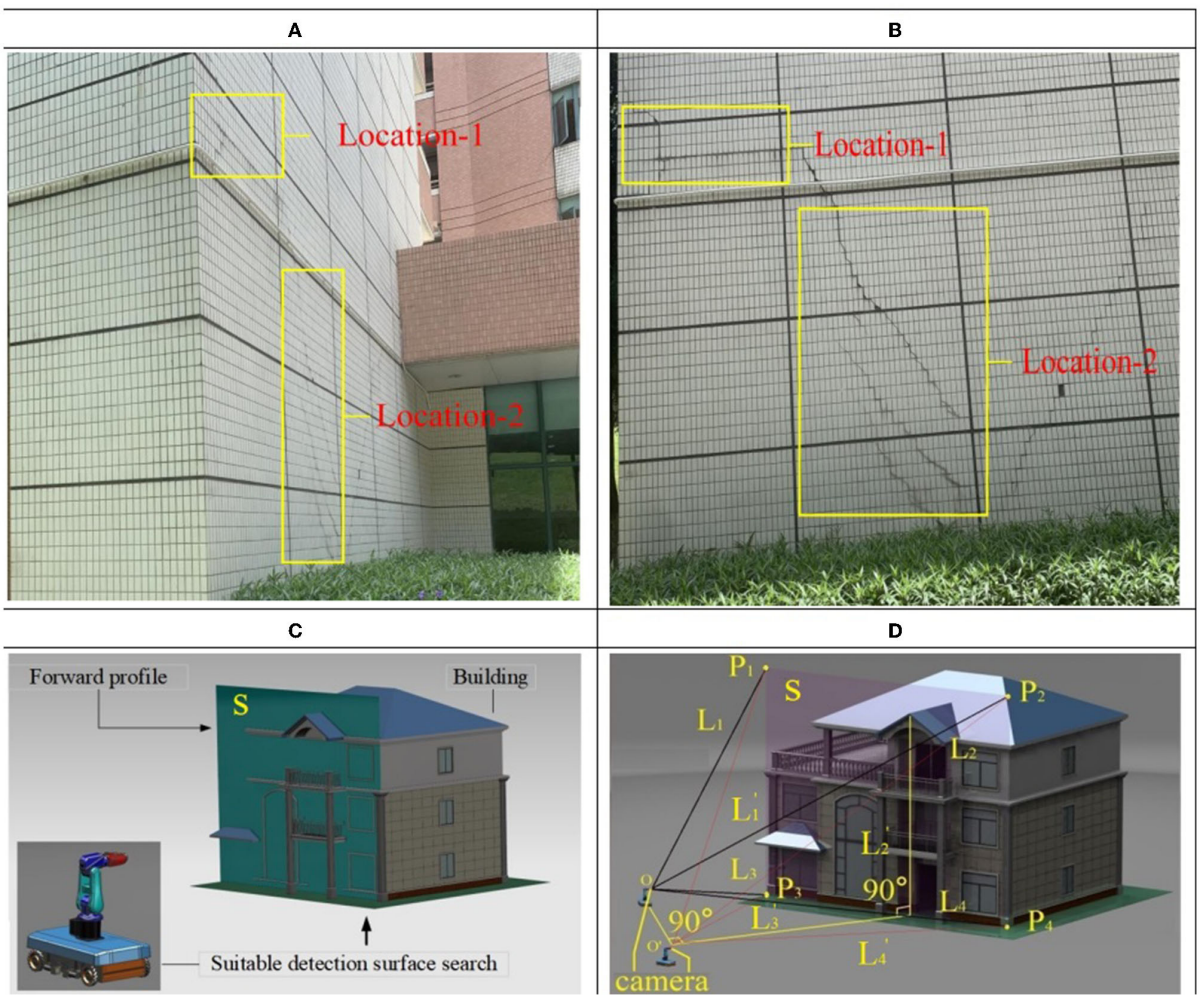
Relationship between camera depth of field and shooting angle. **(A)** Take a photo from the front of the building. **(B)** Take a photo from the side face of the building. **(C)** Shooting angle planning. **(D)** Principle of shooting angle planning.

After the small-step path planning method with MRL, the best robot inspection location will be found. In order to obtain high-quality photos to evaluating the safety of the building, the best shooting angle needs to be found, as shown in Figures 4A,B, two different angled photos of the building in the post-earthquake environment, cracks shown at location-1 and location-2 in photos are different. At the same time, because the oblique angles is different that the clarity of the photo is also different. However, the clear distribution of the cracks is important for evaluating the safety of the buildings. Usually, the photos obtained from the shooting angle of the damaged surface (forward profile) of the building can best reflect the distribution of cracks (as shown in Figure 4C), and the photos are also the clearest, the principle is shown in Figure 4D, the camera in o position is radially offset from the profile S, whereas the camera in o' position is radially perpendicular to the profile S. An appropriate depth of field is the premise of obtaining a satisfactory photo. Under fixed lens parameters, whether a camera can capture a satisfactory photo depends on the appropriate depth of field (the difference between the foreground depth and rear depth of field of a majority of cameras is <1 m). The depth of field is calculated, as shown in Eq. (1).


(1) $$\{\begin{array}{c} \Delta L_{1}=\frac{F\delta L^{2}}{f^{2}+F\delta L}\\ \Delta L_{2}=\frac{F\delta L^{2}}{f^{2}-F\delta L} \end{array}\;\Rightarrow \Delta L=\Delta L_{1}+\Delta L_{2}=\frac{2f^{2}F\delta L^{2}}{f^{4}-\;F^{2}\delta^{2}L^{2}},$$

where δ represents the diameter of the allowable dispersion circle, *f* denotes the focal length of the lens, *F* represents the shooting aperture value of the lens, Δ*L*_1_ denotes the front depth of field, Δ*L*_2_ indicates the rear depth of field, and Δ*L* denotes the depth of field.

The size of the buildings detected in this study are larger than the depth of field (the difference between the foreground depth and the rear depth of field). To obtain clear photos of the building, the adjustment of the angle between the camera and the building (affecting the area of the depth of field) plays an important role when capturing photos. The focal point of the camera is located on the central axis of the building so that the contour of the object is symmetrically distributed. This has a significant impact on the detection results of the displacement, crack, and deformation of the building in a post-earthquake environment. Consider the axial section S of the building as the reference plane. In Figure 4D, L_1_, L_2_, L_3_, and L_4_ are the distances from position o (focal point of the camera) to points P_1_, P_2_, P_3_, and P_4_ of the building, respectively, L_1_', L_2_', L_3_', and L_4_' are the distances from the position o' to the points P_1_, P_2_, P_3_, and P_4_ of the building, respectively. However, L_1_ ≠ L_2_ and L_3_ ≠ L_4_. It can be observed from Eq. (1) that Δ*L* of the two points P_1_ and P_2_ (and P_3_ and P_4_) as in the photo captured by the camera is different, the position o', L_1_' = L_2_', and L_3_' = L_4_', the Δ*L* is same. Considering the inclination of a building in post-earthquake environment, as an example, the different Δ*L* of the left and right sides in the photo will interfere with the corner detection algorithm. Therefore, it is an important prerequisite for the building safety assessment to find the best inspection angle, which is perpendicular and centered to the inclination of the building. The small-step path planning method with MRL proposed in this study can not only be used for unexpected obstacles and unknown disaster avoidance in demanding environments after earthquakes, but also can be used to adjust the robot shooting angle location with small steps of 0.5 m.

### Photos-Based Building Damage Assessment

The Harris algorithm is a corner detection algorithm proposed by Harris and Stephens (Chris and Mike, 1988)). This algorithm has the advantages of achieving speed in calculation and high precision, but it also has the shortcomings of single scale, large amount of calculation, and multiple false corner points. The improved Harris algorithm overcomes the shortcomings of the plain by combining the following three methods: (1) integrating the Gaussian kernel convolutional function to solve the problem of single scale of the Harris algorithm, (2) calculating the gray-scale difference of the initial pixel points to reduce the amount of calculations, (3) using the method of USAN area judgment to improve the accuracy of corner detection. The Gaussian kernel convolutional function method is shown in Eqs. (2) and (3):


(2) $$\begin{array}{r} L\left(x,y,\sigma \right)=G{\left(x,y,\sigma \right)}^{*}I\left(x,y\right) \end{array}$$

(3) $$\begin{array}{r} G\left(x,y,\sigma \right)=\frac{1}{2\pi \sigma^{2}}e^{-\frac{{\left(x-m/2\right)}^{2}+{\left(y-\;\pi /2\right)}^{2}}{2\sigma^{2}}} \end{array}$$

where “^*^” denotes the convolution operation; *m* and *n* denote the size of the Gaussian template; (*x, y*) denotes the pixel point position; and δ indicates the scale parameter. The multi-scale sequence of the photo is obtained by transforming the photo using continuously varying scale parameters.

In the improved algorithm, for a photo size of M × N, the scale space *L*(*x, y*, δ) can be defined as the convolution of the original parameters and Gaussian function *G*(*x, y*, δ).

The Gaussian function can map the information in the original photo to a higher dimension. This method of transforming the scale helps to extract the features of the information in the photo.

**Figure 5:**
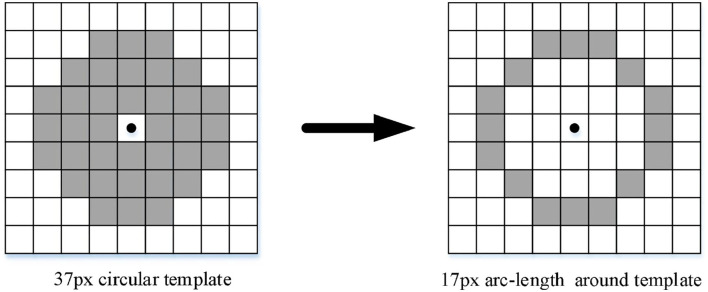
Circular template transform as arc–length template.

Aiming at the shortcomings of Harris, the large amount of calculations need a long time to detect, and 17 pixels arc-length template is used to replace the circular template (37 pixels) to reduce the detection time; the process is shown in Figure 5.

Let the number of pixels similar to it in the neighborhood of the pixel coordinate to be measured as *n*(*x, y*). Then, the formula for the calculation is as follows:


(4) $$C(x,y)=\{\begin{array}{cc} 0 & \forall d_{i}\leq g\\ 1 & \exists d_{i}>g \end{array}(i=1,\;2,\;3,\;4,\;5,\;6,\;7,\;8)$$

(5) $$\begin{array}{c} n(x,y)=\sum (x,y), \end{array}$$

where (*x, y*) is the coordinate value of the target pixel on the template and *g* is the grayscale difference threshold.

## Experiment and Result

An experimental platform was established to verify the designed robot, path planning method, and detection performance. We mainly focus on the verification of the proposed path generation method for an efficient object search, the importance of the correct shooting angle for corner detection, and the effect of our proposed improved Harris algorithm.

### The MRL Method for Robot Path Planning and the Best Camera Shooting Angle

**Figure 6:**
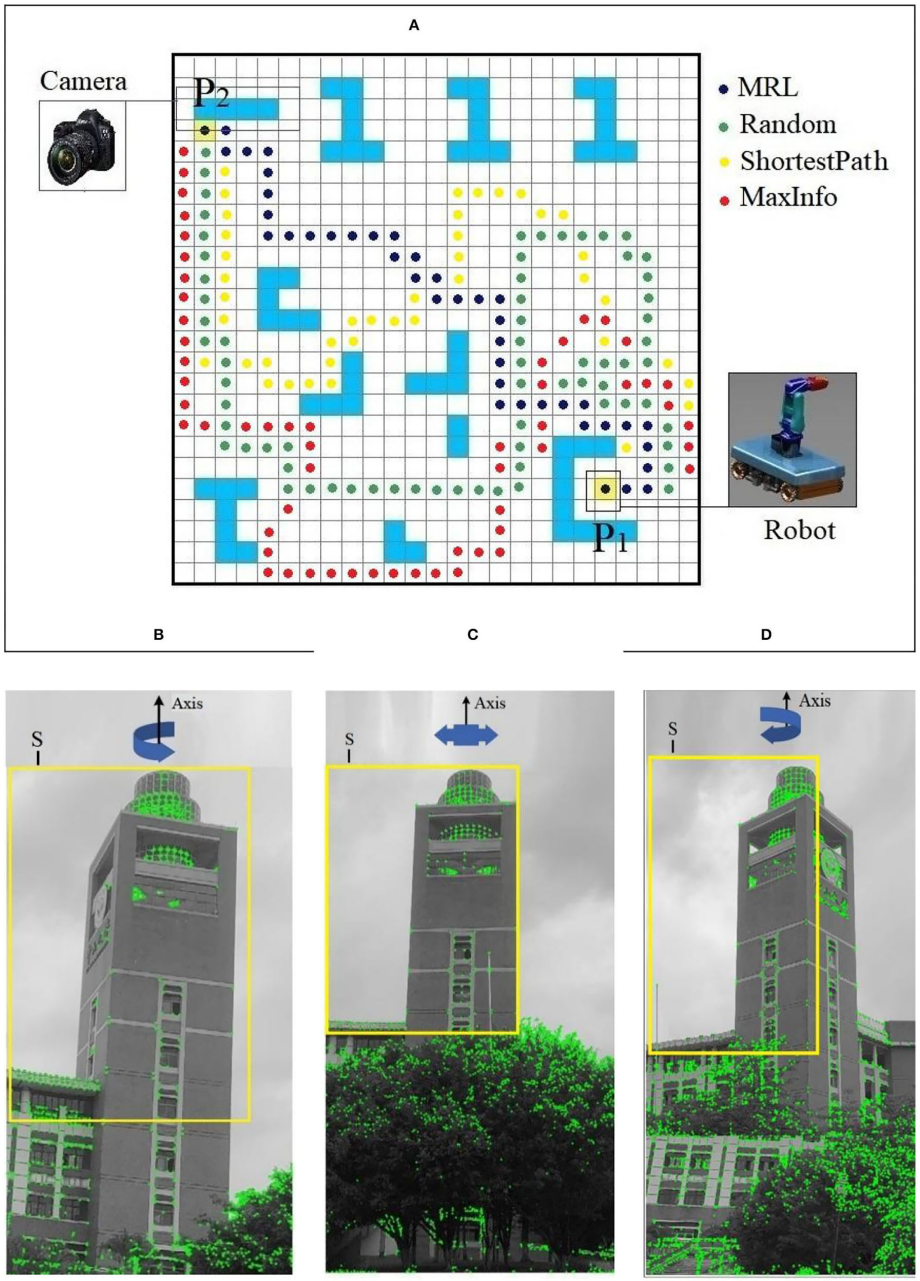
Result of MRL and shooting angle for building safety assessment. **(A)** Comparison of path planning algorithms. **(B)** Shooting angle axis is offset to the left. **(C)** Shooting angle axis is symmetrical. **(D)** Shooting angle axis is offset to the right.

According to the characteristics of obstacles distribution in the post-earthquake environment from starting point A to ending point B, for the shortest distance from point A to point B for robot path planning, we compared our MRL method with the random object search method (Solberg et al., 2008), the MaxInfo method (Kollar and Roy, 2009), and the ShortestPath method (Dornhege and Kleiner, 2013) when the robot reaches position B, take photos from three angles of the target building (axis offset left, axis vertical, and axis offset right), through the corner detection effect to confirm the impact of the shooting angle on the building safety detection, the experimental results are shown in Figure 6.

Figure 6A shows the distribution of obstacles in the post-earthquake environment, which demonstrates that our MRL method can find the target object with less path length than the other methods. The MRL algorithm can make the robot move in the shortest distance from P_1_ to P_2_, it is inspired by human walking behavior and direction decision-making in the post-earthquake environment; it is more suitable for robot path planning to inspect the safety status of the buildings in the post-earthquake environment. Figures 6B–D show the photos taken from different angles when the robot reaches the vicinity of the building after passing the MRL algorithm path, B is the axis offset to the left, C is the axis symmetrical, and D is the axis offset to the right. It can be seen from the formula [Eq. (1)] that C has the most uniform distribution of photo clarity. Through Harris corner detection, it is found that the detection effect of the building core detection area S, as shown in Figure 6C, is the best, which is better than the corresponding ones shown in Figures 6B,D. The regions detected 18% and 20% more ground-truth corners, respectively.

### Effect of Corner Detection Algorithm at Different Shooting Angles

**Figure 7:**
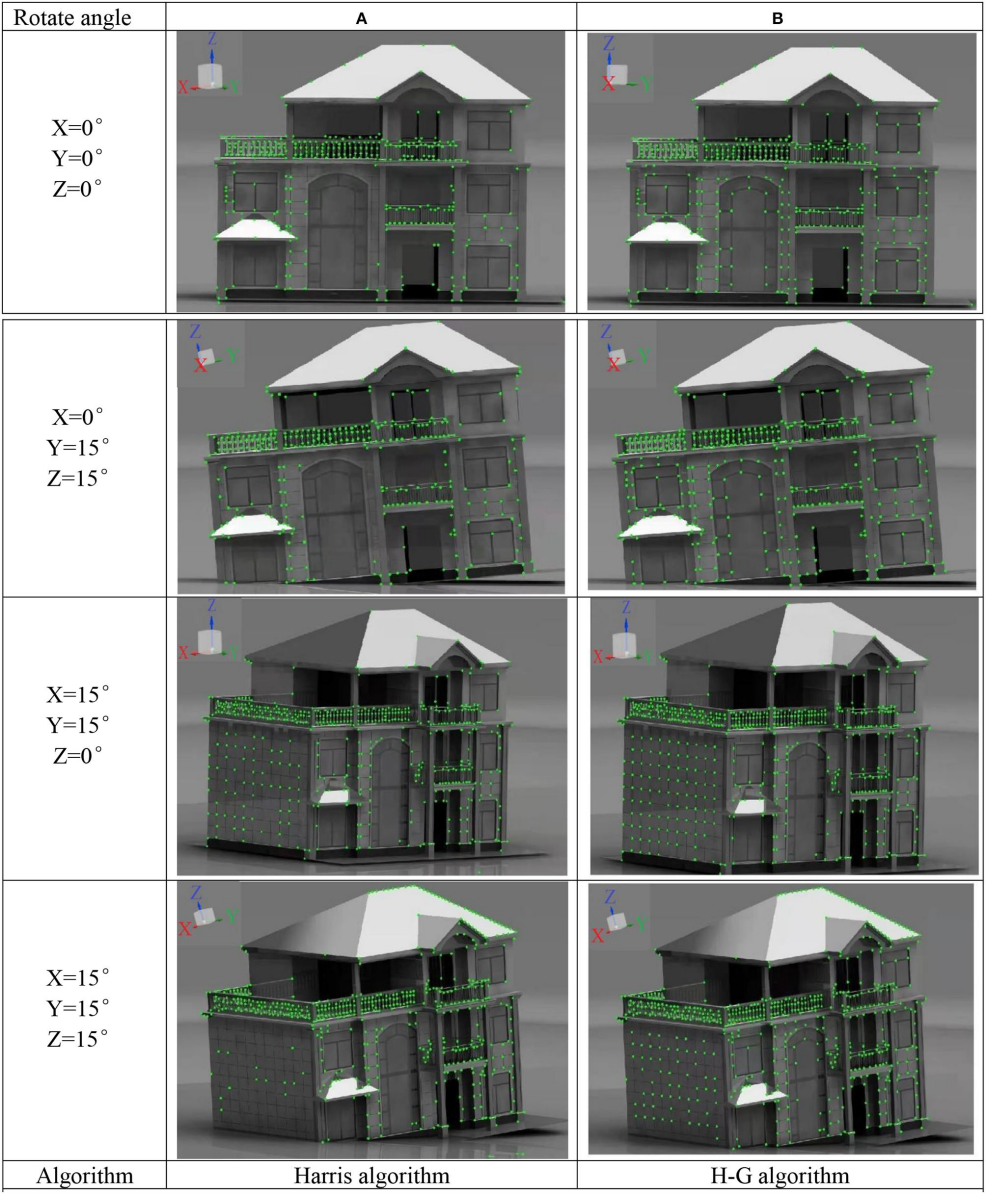
Comparison of corner point detection results before and after improvement. **(A)** Results of corner detection by Harris algorithm. **(B)** Results of corner detection by H-G algorithm.

Taking the photos with axial (X), radial (Y), and tangential (Z) rotate of buildings as objects, the corners of the photos are detected by the improved H–G and Harris algorithm, respectively, the detection results are shown in Figure 7.

Figures 7A,B are showing the corner detection results of the building under different rotation angles by the Harris algorithm and the H–G algorithm, respectively. It can be observed from the photos that at different shooting angles, more real corners were detected by the improved algorithm. The improved detection accuracy was 98%, compared with the plain algorithm, which improved by approximately 20%. The higher corner detection accuracy means that when the building undergoes changes in appearance, such as inclination, displacement, cracks, etc., the algorithm has a more accurate description of the evolution process of corner information after the earthquake (e.g., the generation of cracks is accompanied by the generation of new corners). More accurate results of the corner detection have important reference value for the post-earthquake building safety assessment.

### Improved Corner Detection Algorithms

**Figure 8:**
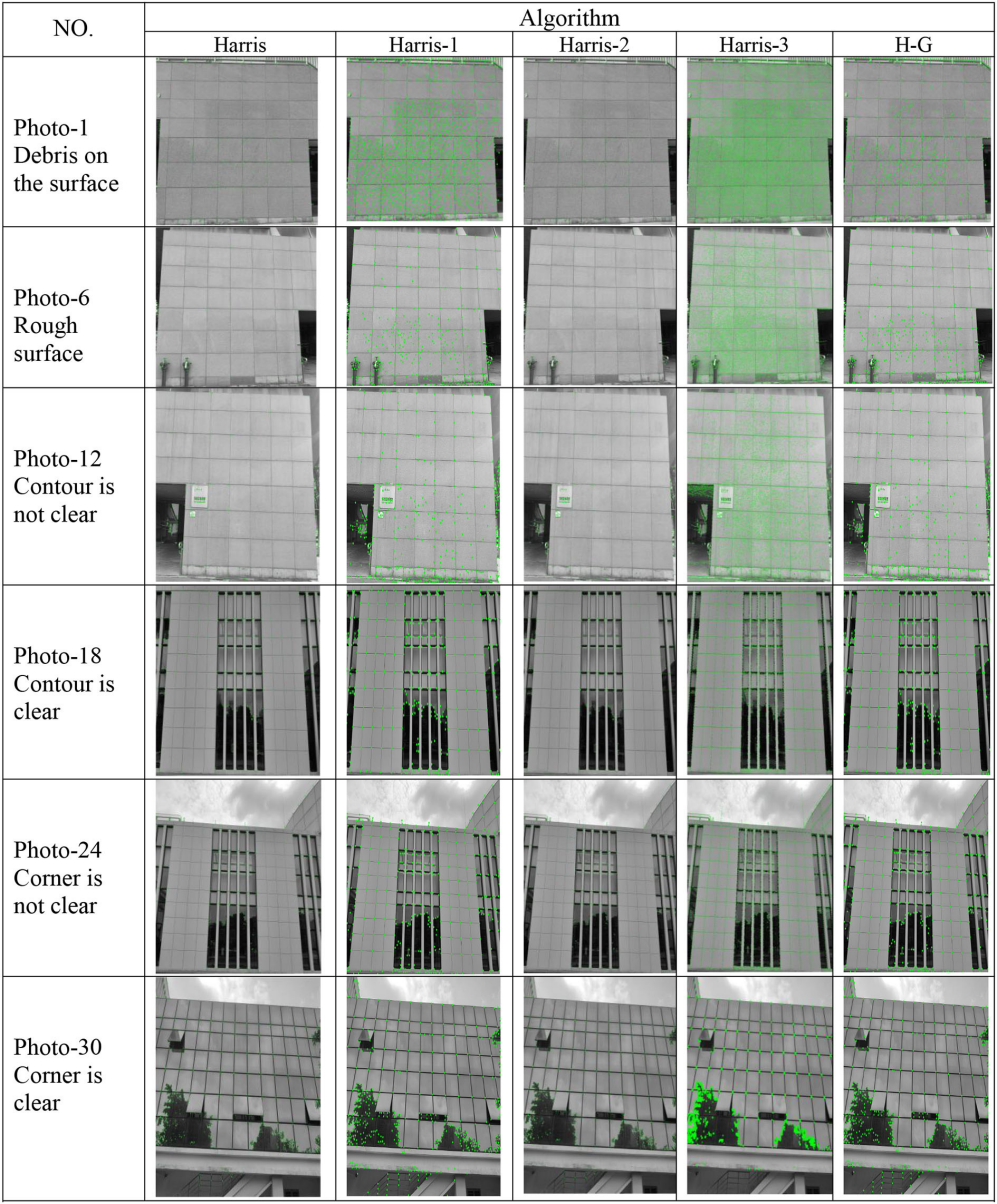
Result of representative photo–class detection.

We performed 30 corner detection photo experiments on 6 types of building appearances on our database using 5 algorithms. In addition, we also compared the effects of the five algorithms through different evaluation indicators. The appearance detection effect of six types of representative buildings is shown in Figure 8, the detection time results of the five algorithms for each picture are shown in Table 1, and the accuracy rate, detection rate, and the average detection time are shown in Table 2.

**Table 1:** Comparison with the average detection time of the five algorithms.

**No**.	**Harris**	**Harris 1**	**Harris 2**	**Harris 3**	**H–G**	**No**.	**Harris**	**Harris 1**	**Harris 2**	**Harris 3**	**H–G**
1	1.1788	0.3152	1.2220	1.2153	0.8181	16	0.7125	0.3051	0.7490	0.6913	0.4866
2	0.8101	0.3088	0.9002	0.8083	0.5629	17	1.3079	0.3243	1.3611	1.3216	0.9585
3	1.1057	0.3269	1.1298	1.2547	0.7194	18	0.5166	0.2939	0.5476	0.5235	0.3647
4	1.4826	0.3679	1.5061	1.5263	0.9992	19	1.0701	0.3430	1.1210	1.0968	0.7862
5	1.3183	0.3489	1.3650	1.2978	0.9430	20	1.6090	0.3430	1.8122	1.6672	1.1530
6	2.6264	0.3291	2.6863	2.6312	1.8362	21	0.9310	0.3353	0.9610	0.9711	0.6579
7	1.2170	0.3556	1.3111	1.2270	0.8330	22	1.9091	0.3660	1.9678	1.8864	1.4001
8	1.3086	0.3455	1.3588	1.2876	0.9624	23	0.7559	0.3217	0.7980	0.7278	0.5153
9	1.3825	0.3286	1.4578	1.3759	0.9714	24	0.6870	0.2931	0.7417	0.6571	0.4769
10	1.3378	0.3618	1.3715	1.3010	0.9617	25	1.3761	0.3349	1.4203	1.3782	1.0258
11	2.3462	0.3322	2.5108	2.3544	1.6776	26	1.0657	0.3467	1.1092	1.0585	0.7788
12	1.1526	0.3605	1.1794	1.1327	0.8496	27	1.5914	0.3604	1.6540	1.5604	1.1473
13	1.3263	0.3525	1.3542	1.3312	0.9650	28	1.0164	0.3193	1.0382	1.0412	0.7087
14	0.7489	0.3444	0.7767	0.7519	0.5109	29	0.8818	0.3216	0.9078	0.9007	0.5743
15	0.6562	0.3028	0.7222	0.6864	0.4580	30	0.9340	0.3091	0.9389	0.9625	0.6507

**Table 2:** The average index value of the five algorithms of this study.

**Algorithms**	**Average**	**Average**	**Average**
	**accuracy (%)**	**detection**	**detection**
		**rate (%)**	**time (s)**
Harris	23.55	75.56	1.2121
Harris 1	39.94	55.23	0.3333
Harris 2	46.76	83.42	1.2660
Harris 3	54.55	83.99	1.2209
H–G	51.89	87.14	0.8584

The 6 types of building appearances in Figure 8 are Debris on the surface (photo 1–5), Rough surface (photo 6–10), Contour is not clear (photo 11–15), Contour is clear (photo 16–20), Corner is not clear (photo 21–25), Corner is clear (photo 26–30) respectively, we select photo1, photo 6, photo 12, photo 18, photo 24, and photo 30 among the 6 types as representative photos, and the five algorithms for detecting photos are Harris, Harris-1, Harris-2, Harris-3, H-G (ours) respectively.

The experimental results show that compared with other algorithms, our proposed algorithm (H–G) can detect more real corners under different building appearances, which is better than the other corner detection algorithms, and compared with the original Harris corner detection algorithm, 20% more real corners are detected by H–G algorithm.

It can be observed from Table 1 that the detection time of the algorithm in Harris 1 (Zheng and Lin, 2020) is the shortest. The edge contour of the photo was extracted using the Canny edge operator to detect the corner of the edge contour. As the number of pixels of the edge contour was far less than the number of pixels in the photo, the efficiency of the algorithm in Harris 2 (Zhong et al., 2010) improved significantly. In Harris 3 (Hou, 2010), the algorithm improves the accuracy of corner detection through the bilateral threshold, which does not involve improving the corner detection speed. However, it increases the calculation of one lower threshold compared with the original Harris algorithm. Thus, the running time is slightly longer than the corner detection time of the original Harris algorithm. The improved algorithm in reference, Harris 3 (Hou, 2010), does not involve improving the response speed of the algorithm, so the detection time is not much different from the original Harris algorithm. In this study, the improved algorithm optimizes the defined 37-pixel circular template by optimizing a 17-pixel circular template, which reduces the amount of calculation, significantly improves the corner detection speed, and reduces the corner detection time. Therefore, compared with the original Harris algorithm and the algorithms mentioned in Harris 2 (Zhong et al., 2010) and Harris 3 (Hou, 2010), the H–G algorithm in this study has the shortest average detection time and the fastest response speed.

The five algorithms discussed in Harris 3 consider the average value of the related indicators of the corner points of the building photos to obtain five algorithm-related indicators, as shown in Table 2.

As shown in Table 2, the Harris algorithm has the lowest accuracy for detecting the corner point in building photos, whereas the other four algorithms have improved in accuracy. In conjunction with the correct rate and detection rate of the different types of building corner detection, the improved algorithm proposed in this study has the best detection effect when compared to other algorithms. Compared to other types of building photo detection, the detection effect also enhances a certain increase. In Harris 1, the Canny operator and Susan operator were combined to detect the angular point of the edge contour of the photo. Although the detection time was reduced significantly, the real angle of the partial photo was lost, resulting in a reduction in the detection rate. In Harris 2, the bilateral threshold was increased based on the original algorithm for the angular point screening, and the accuracy of the algorithm was improved using a small increase algorithm. However, it does not improve the detection speed of the algorithm. In Harris 3, by replacing the center value similar to that in the original algorithm with the center pixel point grayscale value, the region of the center pixel point adjacent to the center pixel point was reduced by the imperial detection and misuse of the original algorithm, thereby improving the accuracy of the algorithm, which was not large compared to the detection time of the algorithm. Therefore, in this study, we improved the algorithm to increase the accuracy of the algorithm, after the angular point detection, mean threshold settings in the multi-scale building image after the wavelet transforms improved the detection speed and the real-time of the algorithm by improving the screening of the ring template.

## Conclusion

To minimize the secondary disasters of earthquakes, in this study, we considered dangerous buildings damaged by earthquakes as the object and proposed a distributed small-step path planning method with MRL to plan a route for the robot in demanding environments. To ensure that the path planning is optimal in a neighboring safe region, a limited distance and 12 directions were gridly refined for the robot to move. When the robot reached the vicinity of the damaged building, it captured a high-quality photo at a suitable camera angle. It used the improved Harris corner detection algorithm to analyze the deformation of the building in the picture for the safety assessment of the building. The experimental results show that the building corner capture accuracy using the proposed method is higher than that of the plain (Harris) corner detection algorithm. However, some problems were also exposed. For example, the robot needs to be in a suitable position so that the focal length of the camera is perpendicular to the building's tilt direction. However, this position is difficult to determine as it can only be approached by visual inspection in 0.5-m steps. Although the optimal position cannot be found theoretically, the experiment shows that the deviation within 0.5 m does not affect the photo quality. After analyzing the parameters of the entire system, we believe that the error of 0.5 m is within the depth of field of the camera. Additionally, with the development of science and technology, especially further breakthroughs in the basic theory of neuro–brain science, the understanding of intelligent expression and decision-making by the people will be improved to a certain extent, thereby enabling the robot to have a more accurate understanding of the scene information to make more intelligent decisions. Thus, new technology will enable a more accurate assessment of building safety.

The aforementioned experiment shows that (1) distributed small-step path planning method with MRL has a shorter path compared with that of the plain random algorithm method; (2) the shooting angle of the camera affects the accuracy of corner detection. In the same structure, the shooting angle radial vertical of the camera of the building detection plane has high (20% improved) accuracy compared with the radial offset of the building detection plane; and (3) the improved Harris algorithm proposed in this study is 20% better than the plain Harris algorithm.

## Data Availability Statement

The raw data supporting the conclusions of this article will be made available by the authors, without undue reservation.

## Author Contributions

ZT: conceptualization, methodology, software, investigation, formal analysis, and writing the original draft. PW: data curation and writing the original draft. YW: visualization and investigation. CW: resources and supervision. YH: conceptualization, funding acquisition, resources, supervision, and reviewing and editing the manuscript. All authors read and edited the manuscript, and agree with its content.

## Funding

The research is financially supported by the National Key R&D Program of China (2021YFC3001000). The project name is key technologies for derivative composite disaster assessment and emergency adapting in the Guangdong–Hong Kong–Macao Greater Bay Area.

## Conflict of Interest

The authors declare that the research was conducted in the absence of any commercial or financial relationships that could be construed as a potential conflict of interest.

## Publisher's Note

All claims expressed in this article are solely those of the authors and do not necessarily represent those of their affiliated organizations, or those of the publisher, the editors and the reviewers. Any product that may be evaluated in this article, or claim that may be made by its manufacturer, is not guaranteed or endorsed by the publisher.
